# Recurrent Cyclic Vomiting and Gastroparesis-Like Symptoms in a Patient With Mast Cell Activation Syndrome (MCAS): A Case of Gastrointestinal-Predominant MCAS

**DOI:** 10.7759/cureus.103302

**Published:** 2026-02-09

**Authors:** Mark Salib, Almir Music, John Salib, Elias Kondilis, Joseph Moss, Sharon Kang

**Affiliations:** 1 Medicine, St. George's University School of Medicine, St. George's, GRD; 2 General Surgery, Washington University of Health and Science, San Pedro, BLZ; 3 General Surgery, Community First Medical Center, Chicago, USA; 4 Internal Medicine, Ross University School of Medicine, St. Michael, BRB; 5 Family Medicine, Humboldt Park Health, Chicago, USA

**Keywords:** cyclic vomiting syndrome, functional gastrointestinal disorders, gastric emptying, gastrointestinal dysmotility, gastroparesis, histamine-mediated disease, hypersensitivity, mast cell activation syndrome, mast cell–mediated disease, neuroimmune gastrointestinal interaction

## Abstract

Mast cell activation syndrome (MCAS) remains an underrecognized cause of gastrointestinal morbidity, frequently presenting with symptoms indistinguishable from functional disorders. Such diagnostic ambiguity can misdirect management and prolong patient symptoms. Thus, we present a case of a 36-year-old male with MCAS who presented with acute, intractable nausea, vomiting, and left-sided back pain. Examination revealed tachycardia and tenderness in the left costovertebral angle. Laboratory evaluation revealed mild anemia, thrombocytopenia, and slightly elevated bilirubin levels. Abdominal and pelvic CT was unremarkable, and upper endoscopy showed mild mucosal erythema with retained gastric contents. Gastric emptying scintigraphy confirmed delayed gastric emptying consistent with mast cell-mediated dysmotility. This case highlights an uncommon gastrointestinal presentation of mast cell activation, emphasizing the importance of considering MCAS in patients with recurrent unexplained vomiting and delayed gastric emptying.

## Introduction

Mast cell activation syndrome (MCAS) is a chronic disorder characterized by the inappropriate and episodic release of mast cell mediators, leading to multisystem symptoms that involve the skin, respiratory tract, cardiovascular system, and gastrointestinal (GI) tract [1-3]. Unlike systemic mastocytosis, MCAS does not require clonal mast cell proliferation or organ infiltration for diagnosis; instead, it is defined by recurrent symptoms consistent with mast cell mediator release, objective biomarker elevation during episodes, and clinical improvement with mediator-targeted therapy [1,4]. While cutaneous flushing, anaphylactoid reactions, and cardiovascular manifestations are well recognized, GI involvement is increasingly reported but remains poorly understood [3,5,6].

GI predominant MCAS can mimic several functional and structural disorders, including irritable bowel syndrome, functional dyspepsia, gastroparesis, and cyclic vomiting syndrome [3,7-9]. Proposed mechanisms include the local effects of histamine, prostaglandins, leukotrienes, and tryptase on intestinal smooth muscle, gastric motility, vascular tone, and visceral afferent signaling [2,5,7]. These mediators may cause nausea, abdominal pain, delayed gastric emptying, and intolerance to high-histamine or high-fat foods [2,10]. However, published cases describing MCAS presenting with recurrent cyclic vomiting and gastroparesis-like delayed gastric emptying remain limited, contributing to diagnostic uncertainty and delayed recognition in clinical practice [3,9].

Recent literature suggests that mast cell-mediated dysmotility may represent a reversible form of gastric transit delay, driven by mediator-induced changes in smooth muscle contractility and enteric nerve signaling, rather than fixed structural disease [5,7,11]. This mechanism may help distinguish MCAS-related episodes from primary gastroparesis, which commonly exhibits persistent symptoms and requires prokinetic therapy.

This case highlights an uncommon GI-dominant flare of MCAS in a patient with longstanding mediator-triggered symptoms. His presentation emphasizes the significance of considering MCAS in patients with recurrent vomiting and routine structural imaging, particularly when symptoms are episodic, food-triggered, and accompanied by flushing or multisystem complaints [1-3]. Similarly, this report contributes to the growing literature describing mast cell mediator-related dysmotility as a reversible cause of delayed gastric emptying [2,7,11].

Early recognition of this phenotype is clinically relevant, as patients often respond rapidly to H1/H2 blockers and mast cell stabilizers, thereby mitigating the need for unnecessary invasive testing or prolonged empirical treatments for idiopathic gastroparesis [2,4,7].

## Case presentation

A 36-year-old male with a past medical history significant for hypertension, asthma, unshunted hydrocephalus, compressed lumbar disc, osteonecrosis of the right hip, polycythemia vera, and MCAS with recurrent anaphylactic episodes presented to the emergency department with acute-onset intractable nausea, vomiting, and left-sided back pain. He reported that his current episode closely resembled prior MCAS flares. His allergies included trimethoprim-sulfamethoxazole, nuts, dairy, methylprednisolone (Solu-Medrol), and shellfish. He denied tobacco, alcohol, and illicit drug use.

The patient had presented to the same emergency department previously in December 2024 with comparable symptoms, during which an abdominal and pelvic computed tomography (CT) imaging was unremarkable. Since that visit, he had experienced progressive worsening of cyclic vomiting, diffuse abdominal discomfort, and increasing food sensitivities. He described intolerance to dairy and high-fat foods, which invariably triggered the episodes of nausea, flushing, and abdominal pain within minutes of ingestion. His symptoms had previously been attributed to irritable bowel syndrome and functional dyspepsia, and empiric treatment with proton pump inhibitors, antiemetics, and dietary modifications had provided minimal alleviation.

Twelve hours prior to his admission to the emergency department, he developed recurrent, non-bloody, non-bilious vomiting exceeding 15 episodes, associated with diffuse abdominal pain and localized left flank discomfort. The flank pain was described as sharp, constant, and non-radiating, with an intensity rating of 5/10, occasionally shifting toward the left upper quadrant. He denied chest pain, shortness of breath, visual changes, urinary symptoms, constipation, or recent trauma.

On examination, the patient was awake, alert, and not in acute distress. He was afebrile (37.3°C) with a heart rate of 108 bpm, blood pressure of 148/92 mmHg, respiratory rate of 20 breaths per minute, and oxygen saturation of 98% on room air. The head was normocephalic and atraumatic, with anicteric sclerae and normal conjunctivae; the neck was supple with a full range of motion. Cardiopulmonary examination was unremarkable, with clear vesicular breath sounds bilaterally and a regular heart rhythm without murmurs, rubs, or gallops. The abdomen was soft and non-distended, with mild tenderness noted over the left flank and left costovertebral angle. No guarding, rebound, or hepatosplenomegaly was appreciated. The extremities showed a full range of motion without edema, although scattered ecchymoses were noted on the upper extremities, which the patient attributed to a prior venipuncture. The skin was dry without urticaria or flushing, and neurologic examination was within standard limits.

Laboratory evaluation (Table 1) revealed a normal white blood cell count (10.1 × 10⁹/L) with relative neutrophilia (82.1%) and lymphopenia (8.2%), mild normocytic anemia (hemoglobin = 12.6 g/dL, hematocrit = 37.5%), and thrombocytopenia (103 × 10⁹/L). The basic metabolic panel demonstrated normal electrolytes and renal function, with a mildly elevated blood urea nitrogen (BUN)-to-creatinine ratio (24.7), consistent with dehydration secondary to the episodes of vomiting. Liver enzymes were within normal limits, though total bilirubin was slightly elevated (1.1 mg/dL). Lipase levels were low (13 U/L), and calcium, magnesium, and glucose levels were within the normal range. These findings were consistent with mild hypovolemia in the setting of acute emesis, with baseline hematologic changes reflecting his underlying polycythemia vera and known mast cell-related marrow involvement.

**Table 1:** Laboratory findings on admission. Initial laboratory evaluation demonstrated mild cytopenias with otherwise stable metabolic and hepatic function. The workup included a complete blood count (CBC), basic metabolic panel (BMP), and liver function tests (LFTs). Mild anemia and thrombocytopenia were noted, consistent with the patient’s underlying hematologic history, while electrolytes, renal indices, and liver enzymes were within normal limits.

Laboratory test	Analyte	Result	Reference range
Complete blood count (CBC)	White blood cell count (WBC)	10.1 ×10⁹/L	3.8–10.8 ×10⁹/L
	Neutrophils (%)	82.1	46–75
	Lymphocytes (%)	8.2	20–45
	Monocytes (%)	9.3	4–8
	Hemoglobin (Hgb)	12.6 g/dL	13.2–17.1 g/dL
	Hematocrit (Hct)	37.50%	38.5–50.0%
	Mean corpuscular volume (MCV)	84.8 fL	80–100 fL
	Platelet count (Plt)	103 ×10⁹/L	140–400 ×10⁹/L
Basic metabolic panel (BMP)	Sodium (Na)	137 mmol/L	136–144 mmol/L
	Potassium (K)	4.2 mmol/L	3.6–5.1 mmol/L
	Chloride (Cl)	102 mmol/L	98–108 mmol/L
	Bicarbonate (CO₂)	24 mmol/L	22–32 mmol/L
	Blood urea nitrogen (BUN)	21 mg/dL	8–26 mg/dL
	Creatinine (Cr)	0.85 mg/dL	0.64–1.30 mg/dL
	BUN/creatinine ratio	24.7	12–20
	Glucose	71 mg/dL	60–100 mg/dL
	Calcium (Ca)	8.5 mg/dL	8.5–10.1 mg/dL
	Magnesium (Mg)	1.9 mg/dL	1.8–2.5 mg/dL
Liver function tests (LFTs)	Total bilirubin (Tbili)	1.1 mg/dL	0.2–1.0 mg/dL
	Aspartate aminotransferase (AST)	38 U/L	15–41 U/L
	Alanine aminotransferase (ALT)	38 U/L	14–54 U/L
	Alkaline phosphatase (ALP)	75 U/L	32–140 U/L
	Total protein (TP)	7.5 g/dL	7.0–9.0 g/dL
	Albumin (Alb)	3.9 g/dL	3.5–5.0 g/dL
Other tests	Lipase	13 U/L	73–393 U/L
	Glomerular filtration rate (GFR, calculated)	116 mL/min	>60 mL/min

**Figure 1:**
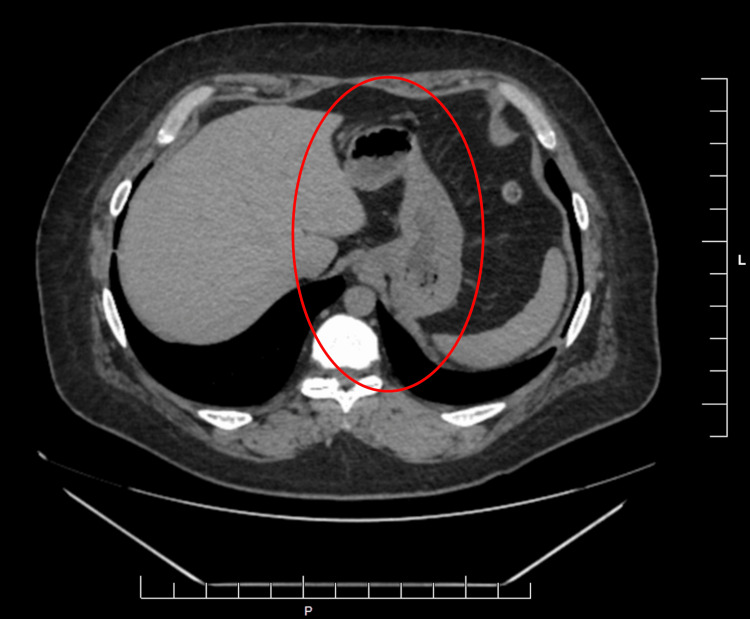
Axial contrast-enhanced computed tomography (CT) of the abdomen and pelvis. The axial image at the level of the gastric body demonstrates mild gastric distension with a visible air-fluid level (as depicted by the red circle). There is no evidence of focal mural thickening, suspicious masses, or mechanical gastric outlet obstruction. Surrounding inflammatory changes, such as fat stranding or free fluid, are notably absent.

**Figure 2:**
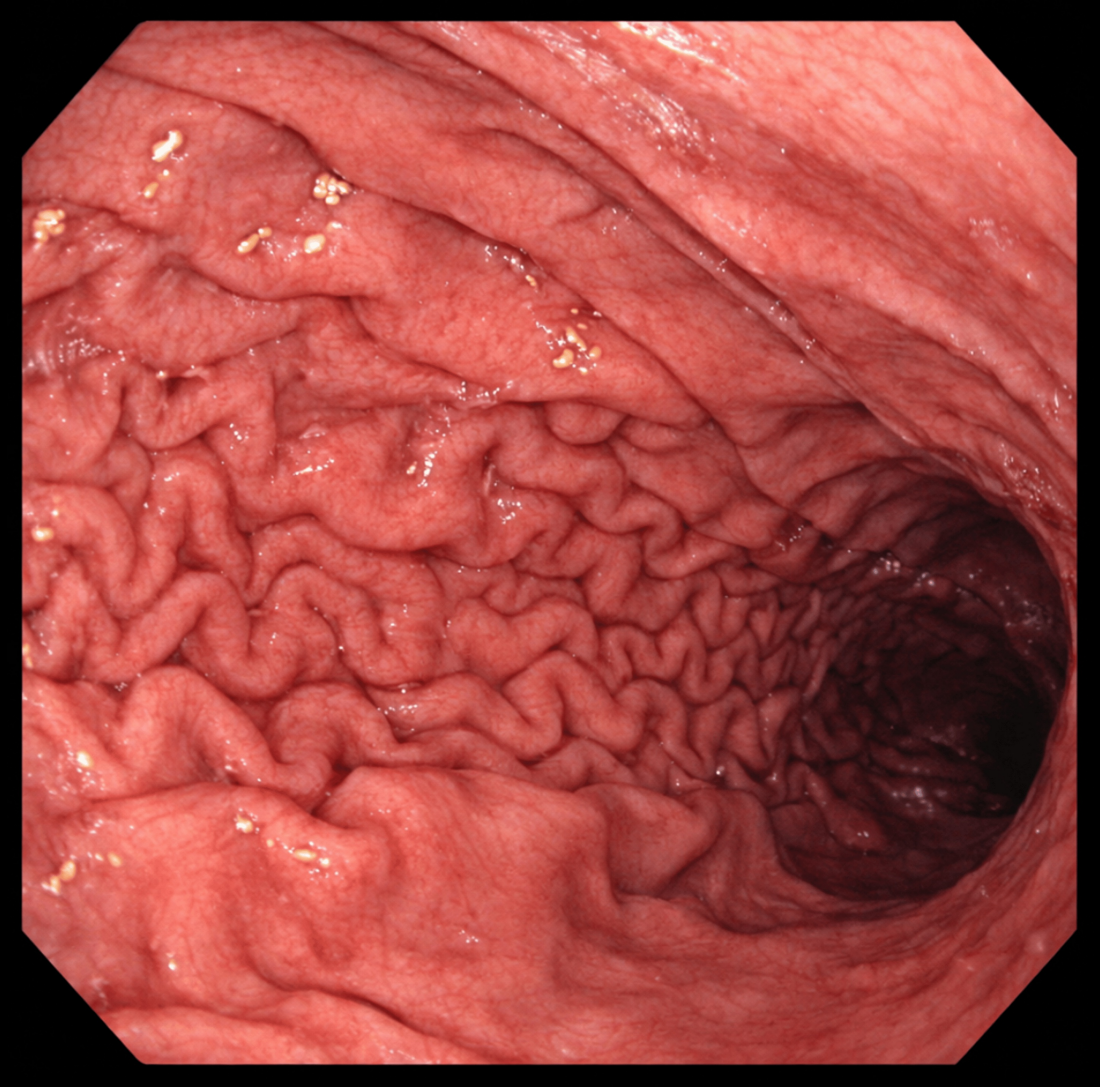
Esophagogastroduodenoscopy (EGD) demonstrating normal gastric mucosa. High-resolution endoscopic image of the stomach demonstrating normal gastric mucosa. The mucosal surface appears smooth and pink with intact rugal folds, a uniform vascular pattern, and no evidence of erythema, erosion, ulceration, nodularity, masses, or lesions.

**Figure 3:**
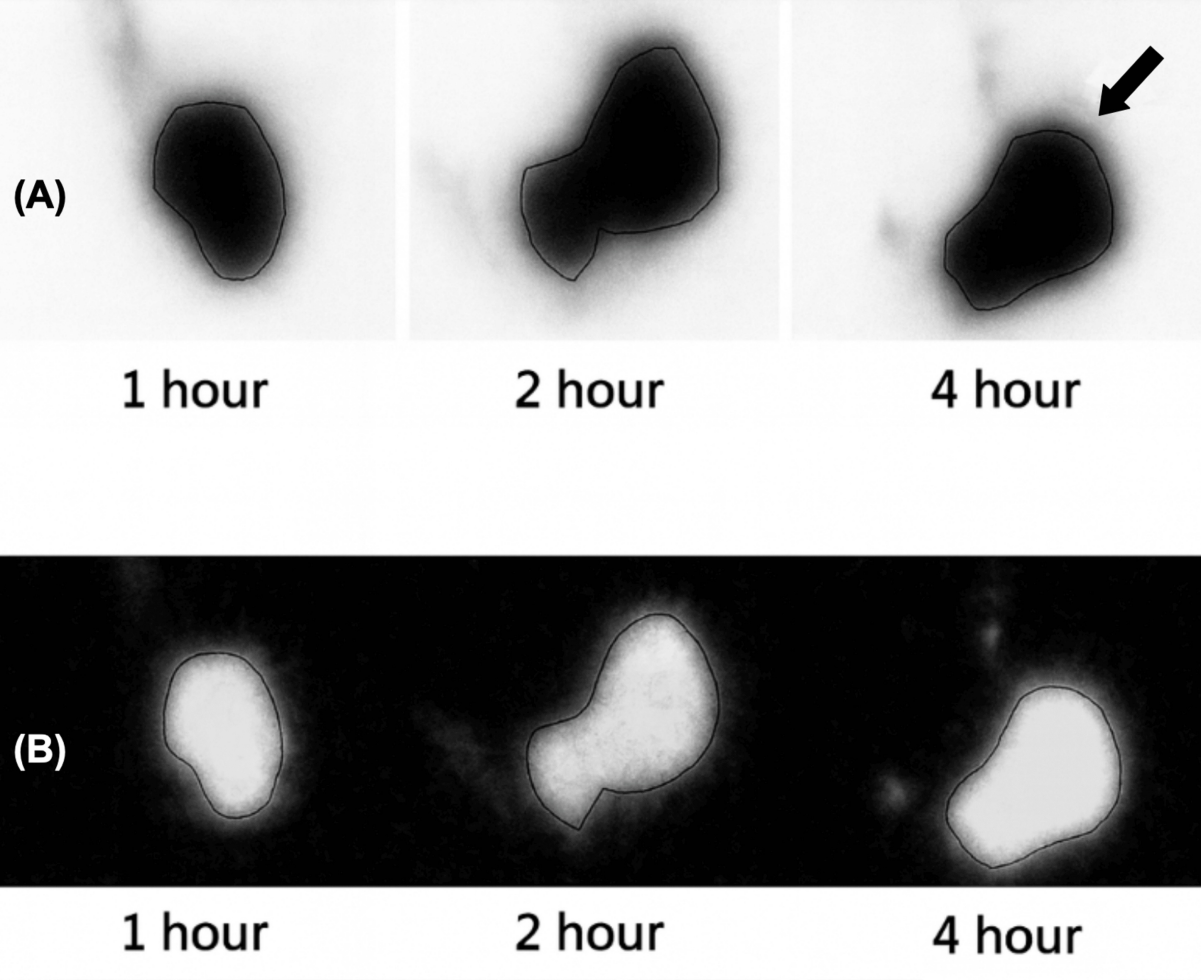
Serial gastric emptying scintigraphy. (A) Anterior gastric emptying images obtained at one, two, and four hours demonstrating progressive but incomplete clearance of radiotracer from the stomach. Persistent intragastric activity at four hours (arrow) exceeds expected normative retention thresholds, consistent with delayed gastric emptying. (B) Posterior gastric emptying images obtained at one, two, and four hours demonstrating delayed radiotracer clearance, supporting impaired gastric transit. These imaging findings align with mast cell-mediated dysmotility in the clinical context of mast cell activation syndrome.

A contrast-enhanced CT of the abdomen and pelvis (Figure 1) demonstrated mild gastric distension without evidence of obstruction, mass, or inflammatory process. Upper endoscopy revealed retained gastric contents and mild mucosal erythema but no ulceration or structural abnormality (Figure 2). Subsequent gastric emptying scintigraphy was obtained (Figure 3) and revealed delayed gastric emptying, consistent with a gastroparesis-like pattern, likely secondary to the effects of mast cell mediators on gastrointestinal smooth muscle.

During his hospital course, the patient was managed supportively with intravenous fluids, antiemetics, and H₂ and H₁ antihistamines for symptomatic control. The patient's home Dilaudid regimen was continued for breakthrough pain, and proton pump inhibitor therapy was maintained. Given his history of MCAS, the patient was also administered cromolyn sodium and montelukast to mitigate mast cell mediator release. Over the next 72 hours, his nausea and emesis gradually improved, and he was able to tolerate clear liquids, followed by a regular diet without recurrence of symptoms. Serial abdominal examinations remained benign, and his hemodynamic parameters stabilized. Laboratory values trended toward baseline, with improvement in hydration markers. He was discharged home on hospital day three in stable condition with instructions for dietary modification, continuation of his mast cell stabilizing regimen, and close outpatient follow-up with gastroenterology and immunology for ongoing management of mast cell-associated gastrointestinal dysmotility.

## Discussion

This patient’s presentation illustrates a gastrointestinal-predominant exacerbation of MCAS, characterized by recurrent, non-bilious vomiting, abdominal discomfort, flushing, and reproducible sensitivity to dairy and high-fat foods. Although vomiting in MCAS is well described [1,3,4], the combination of cyclic episodes, intolerance to high-frequency emesis, and scintigraphy-confirmed delayed gastric emptying resembles a gastroparesis-like syndrome mediated not by structural disease but by mast cell-derived bioactive mediators [2,5,7,11]. This pattern has been increasingly recognized in mediator-driven dysmotility, particularly when symptoms resolve rapidly with antihistamine therapy.

Mast cells reside throughout the gastrointestinal mucosa and submucosa, where activation results in the release of histamine, prostaglandin D₂, leukotrienes, platelet-activating factor, tryptase, and cytokines. Several of these mediators directly impair gastric and intestinal motility [3,4,6,8,9,11]. Histamine acting on H1 and H2 receptors alters smooth muscle contractility and delays gastric emptying [2,10], while prostaglandin D₂ and leukotrienes increase visceral hypersensitivity and contribute to nausea and cramping [2,5,7,12]. Tryptase activates protease-activated receptors (PAR-2), disrupting enteric neuronal signaling [5,7]. This biochemical dysfunction provides a plausible mechanistic explanation for mediator-triggered dysmotility in MCAS, despite unremarkable imaging and endoscopy [2,3]. The patient’s intolerance to high-fat and high-histamine foods further supports an acute mediator-release mechanism underlying his gastrointestinal symptoms [10].

This patient’s documented hypersensitivity to methylprednisolone (Solu-Medrol) is clinically significant, particularly in the context of MCAS, where corticosteroids are often used as first-line therapy during acute flares. Hypersensitivity reactions to methylprednisolone have been reported and may occur through IgE-mediated mechanisms, non-IgE-mediated mast cell activation, or reactions to formulation excipients such as polyethylene glycol, sulfites, or benzyl alcohol [8,10]. Succinate ester formulations, in particular, have been associated with increased immunogenicity [7]. Importantly, cross-reactivity among corticosteroids is not universal, underscoring the need to distinguish methylprednisolone from other glucocorticoids such as prednisolone when documenting allergies and selecting alternative therapies in MCAS patients [10].

**Figure 4:**
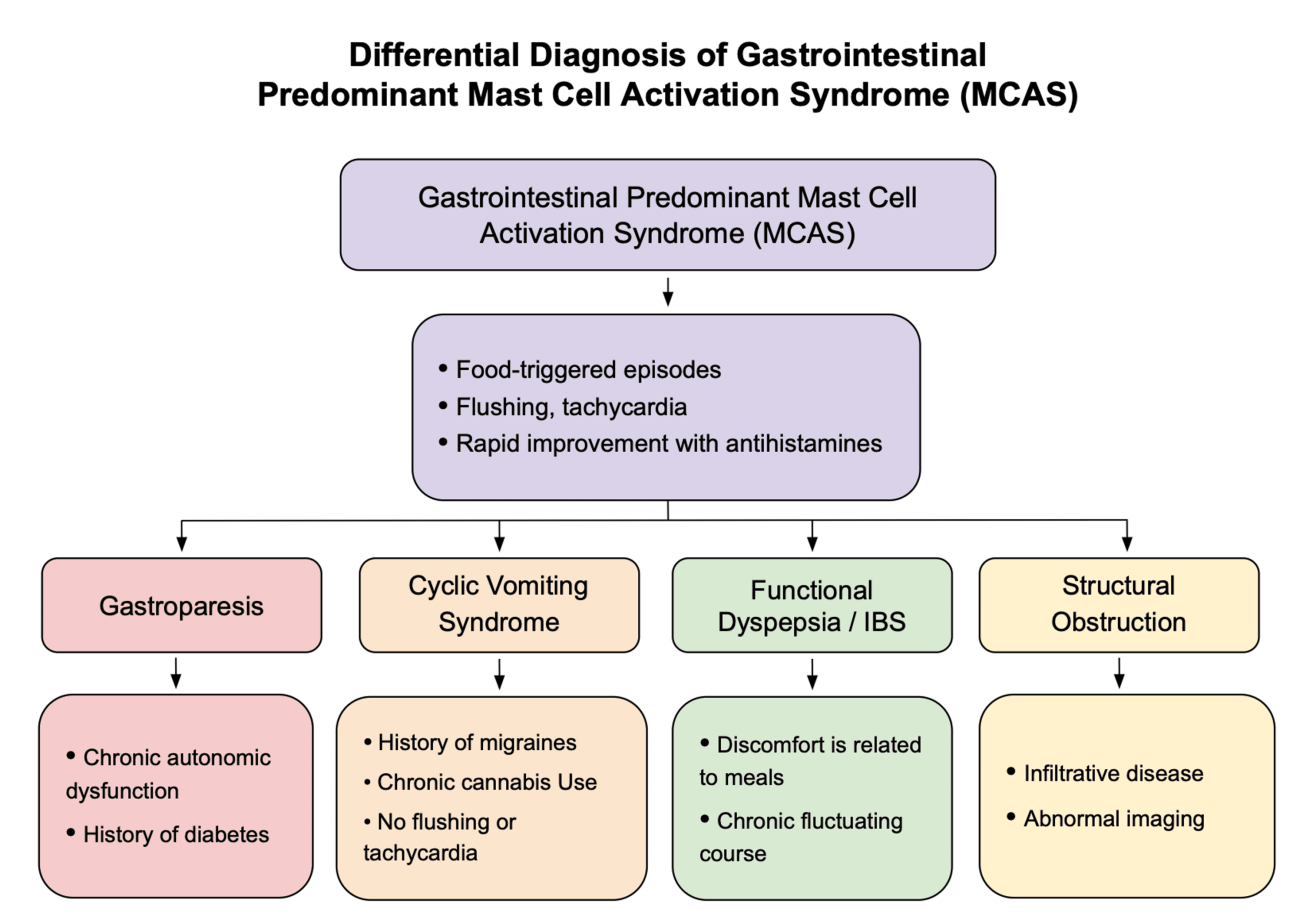
Differential diagnoses in gastrointestinal-predominant MCAS. This flowchart summarizes the primary diagnostic considerations for evaluating gastrointestinal-predominant MCAS. The top tier outlines features suggestive of MCAS, including food-triggered episodic symptoms with flushing, tachycardia, and rapid response to antihistamines [1,7]. The second tier presents key alternative diagnoses: gastroparesis, typically associated with diabetes or autonomic dysfunction; cyclic vomiting syndrome, linked to migraine disorders or chronic cannabis use; functional dyspepsia and irritable bowel syndrome, which produce chronic meal-related symptoms without structural disease; and structural obstruction, indicated by fixed anatomical abnormalities or infiltrative processes on imaging [1,7]. MCAS: mast cell activation syndrome; IBS: irritable bowel syndrome. Figure created and designed by the authors.

Diagnostic complexity in this case stems from overlapping features with several gastrointestinal disorders, including gastroparesis, which is typically associated with diabetes, postsurgical changes, or medication effects. However, this patient demonstrated no structural abnormalities, and imaging was routine [11,13]. Cyclic vomiting syndrome (CVS) shares episodic vomiting but is more commonly linked to migraine, cannabis use, or mitochondrial disorders, none of which were present here [9]. Functional dyspepsia and irritable bowel syndrome generally produce more chronic or meal-related discomfort rather than the patient’s sharply episodic, food-triggered flares [3,6,8]. Additionally, repeated routine CT imaging and standard upper endoscopy argue strongly against obstruction or infiltrative disease. Collectively, as depicted in Figure 4, these entities represent key components of the differential diagnosis in patients with recurrent unexplained vomiting, underscoring the need for careful exclusion before attributing symptoms to mast cell-mediated pathology.

The patient’s recurrent flushing, food-triggered symptoms, standard imaging, and dramatic response to antihistamines and mast-cell stabilizers favor MCAS as the primary etiology [1,2,4]. These distinguishing features also favor MCAS over primary motility disorders, particularly given the reversible nature of the gastric emptying delay [2,5,7,11].

**Figure 5:**
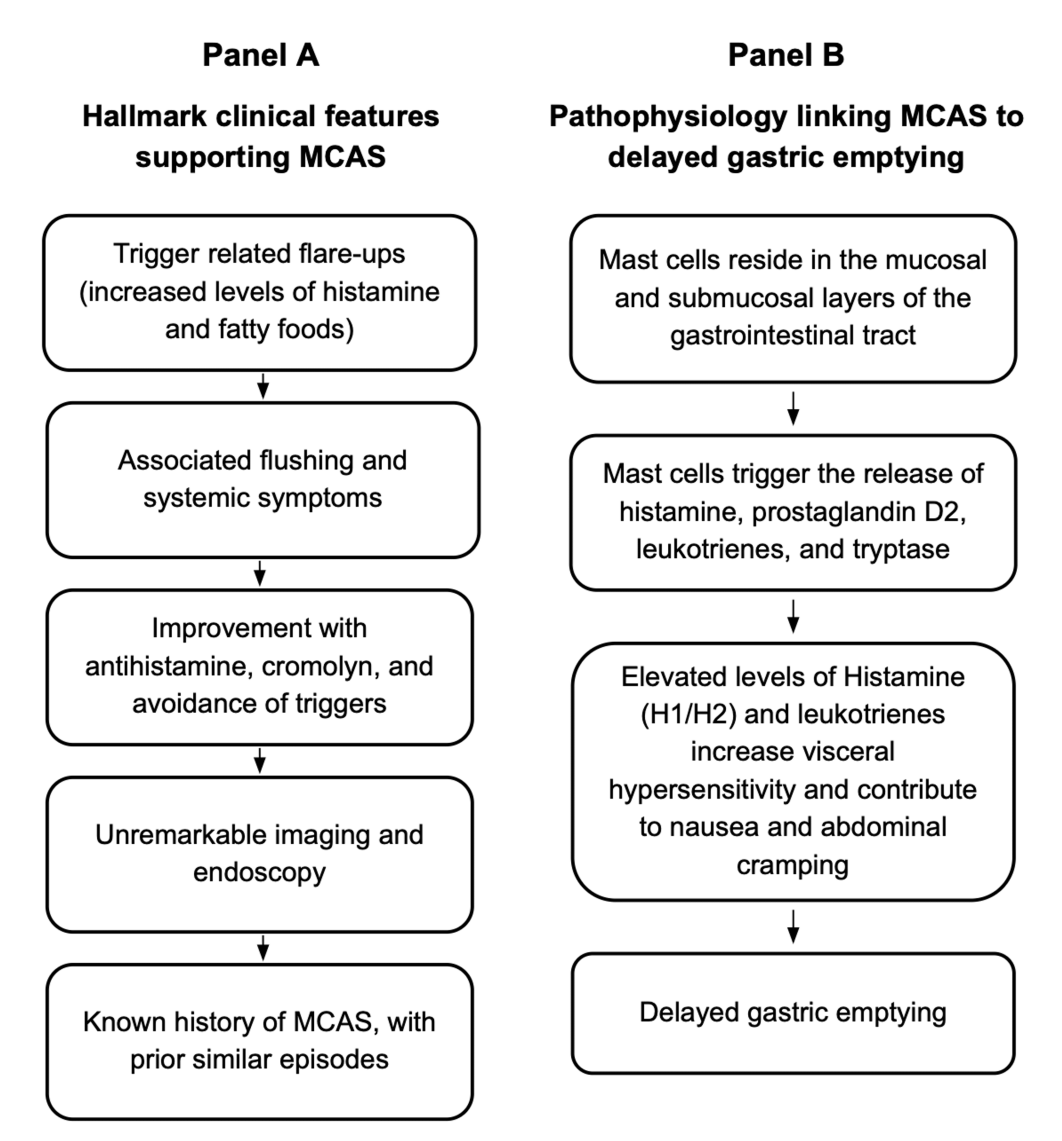
Diagnostic flowchart for gastrointestinal-predominant MCAS. This flowchart outlines the stepwise reasoning used to identify mast cell activation syndrome (MCAS) as the cause of the patient’s recurrent vomiting and delayed gastric emptying. The algorithm emphasizes the patient’s episodic symptoms, including flushing and tachycardia, reproducible food-triggered flares, normal structural evaluation on CT and endoscopy, and rapid improvement with antihistamines and mast cell-directed therapy. Together, these findings support gastrointestinal-predominant mast cell activation rather than gastroparesis, cyclic vomiting syndrome, or structural gastrointestinal pathology, in alignment with current diagnostic frameworks [1-3]. Figure created and designed by the authors.

Although this patient had a history of recurrent anaphylactoid episodes, which initially raised concern for systemic mastocytosis. Systemic mastocytosis is defined by clonal mast-cell proliferation and tissue infiltration, meeting the WHO criteria that include multifocal dense mast-cell aggregates on biopsy, a KIT D816V mutation, aberrant expression of CD25 or CD2, and a persistent elevation of baseline serum tryptase [1]. None of these features was present in this patient. Endoscopic biopsies demonstrated no mast cell infiltration, abdominal imaging was unremarkable, and no clonal markers or elevated baseline tryptase levels were documented. In contrast, MCAS is characterized by episodic release of mast-cell mediators without underlying clonal expansion. The patient’s reproducible food-triggered symptoms, including flushing, tachycardia, cyclic vomiting, delayed gastric emptying, and rapid improvement with H1/H2 blockade and mast cell stabilizing therapy, are more consistent with mediator-driven disease [2-4]. The absence of structural abnormalities and normal biopsy findings further support MCAS as the underlying process rather than systemic mastocytosis. This distinction (as depicted in Figure 5) is clinically meaningful, as prognosis, management strategies, and need for long-term surveillance differ substantially [1,4].

The patient’s symptoms improved rapidly with intravenous fluids, antiemetics, H1/H2 blockade, and continuation of his mast cell-directed medications, including cromolyn and montelukast. This response pattern is consistent with mast cell-mediated dysmotility rather than primary gastroparesis, which typically requires prokinetic therapy and does not resolve within 48-72 hours [11,13]. Importantly, gastric emptying scintigraphy demonstrated delayed gastric transit, supporting prior reports that mast cell mediators can transiently impair gastric motility in susceptible individuals [2,5,7]. This pattern has been described in small case series but remains underrecognized. The rapid reversal of dysmotility following mediator blockade further reinforces MCAS as the mechanism rather than an intrinsic motility disorder [2,7,11]. Emerging work has shown that prostaglandin D₂ and histamine signaling may modulate vagal pathways and enteric neuronal activity, providing a mechanistic link between mast cell activation and episodic vomiting patterns [5,7,11].

Growing evidence suggests that mast cell activation plays a broader role in gastrointestinal dysmotility than previously appreciated. Several studies have identified increased mast cell density or activation markers in patients with functional dyspepsia, non-ulcer dyspepsia, and idiopathic gastroparesis [1,3,8,10,14]. Case reports and small series describe patients with MCAS presenting with nausea, abdominal pain, diarrhea, and delayed gastric emptying [6,15]. However, recurrent cyclic vomiting mimicking CVS remains rare [16], underscoring the clinical relevance of this case. Early recognition of GI-predominant MCAS can prevent diagnostic delays, reduce unnecessary invasive testing, and avoid empiric trials of prokinetics or migraine-directed therapies that are unlikely to resolve mediator-driven symptoms [2,4]. Identifying MCAS early also helps distinguish it from idiopathic gastroparesis or functional GI disorders, reducing misdiagnosis and optimizing therapeutic outcomes [3,8].

## Conclusions

Gastrointestinal manifestations of mast cell activation syndrome are common but frequently underrecognized, particularly when presenting with cyclic vomiting and delayed gastric emptying that resemble gastroparesis or functional vomiting disorders. This case illustrates how the release of mast cell mediators can transiently impair gastric motility and lead to recurrent, food-triggered vomiting, despite routine imaging and structural evaluation. Early recognition of GI-dominant MCAS is crucial, as targeted therapy with antihistamines, leukotriene inhibitors, and mast cell stabilizers can result in rapid symptom resolution. Recognition of cases like this one requires close collaboration between gastroenterology and immunology, which enables a tailored, mediator-directed therapy that leads to rapid and complete resolution of symptoms. Additionally, this case adds to the limited literature describing transient gastric dysmotility directly linked to mast cell activation. High index of suspicion for MCAS in patients with episodic, foot-triggered vomiting accompanied by flushing, tachycardia, or other systemic manifestations, even when standard imaging is unremarkable, should be maintained by clinicians. As the spectrum of MCAS-related gastrointestinal manifestations continues to expand, additional research is needed to clarify the mechanistic pathways and guide evidence-based management strategies for patients with episodic dysmotility.
